# Les ruptures traumatiques du tendon quadricipital: à propos de 3 cas

**DOI:** 10.11604/pamj.2015.22.343.8272

**Published:** 2015-12-10

**Authors:** Youssef Benyass, Bouchaib Chafry, Kaldadak Koufagued, Salim Bouabid, Belkacem Chagar

**Affiliations:** 1 Service de Traumatologie-Orthopédie II, Hôpital Militaire d'Instruction Mohamed V, Rabat, Maroc

**Keywords:** Tendon quadricipital, rupture, chirurgie, quadriceps tendon, rupture, surgery

## Abstract

Les ruptures traumatiques du tendon quadricipital sont rares, elles surviennent préférentiellement après 40 ans, suite à un traumatisme indirect chez le sportif (flexion contrariée du genou) ou traumatisme banal chez le sédentaire. La tendinopathie préexistante est fréquente. La rupture est le plus souvent totale et siège au corps du tendon 60% des cas ou décallotement quadricipital au bord supérieur de la rotule (40% des cas). Le diagnostic est essentiellement clinique. Les examens complémentaires (échographie et imagerie par résonance magnétique) sont utiles et appuient le diagnostique, mais sont souvent faussement rassurants hormis la radiographie qui montre une rotule basse. Le traitement essentiellement chirurgical associé à la rééducation fonctionnelle donne des résultats largement meilleurs. Le délai d'intervention est un facteur pronostic très important. Les auteurs rapportent 03cas de rupture de tendon quadricipital. L’âge moyen est de 50ans. Ils ont été traités chirurgicalement et revus régulièrement, avec un recul de 16 mois pour apprécier l’évolution. Les résultats ont été très bons chez 02 cas et bon chez 01 cas. L'amélioration a été très nette selon les critères: marche, douleur et reprise d'activité physique.

## Introduction

Les traumatismes de l'appareil extenseur du genou correspondent à une solution de continuité qui interrompt la chaîne de transmission de l'extension de la jambe sur la cuisse. Ils comprennent les fractures de la patella et les lésions de l'appareil musculo-tendineux (tendon rotulien et tendon quadricipital). La rupture du tendon quadricipital est une affection rare, environ 2 à 3 cas par an [1, 2]. Elles surviennent préférentiellement chez le sportif après 40 ans suite à un traumatisme indirect ou chez des sujets sédentaires à la suite d'un traumatisme minime. Le diagnostic de cette pathologie passe souvent inaperçu du fait de la méconnaissance ou de la sous-estimation fréquente de ces lésions, et de la structure particulière du tendon qui est lamellaire et constitué de quatre faisceaux. Le retard du diagnostic favorise l'installation d'hypertrophie et des calcifications, source de douleur. L'examen clinique est essentiel. L'imagerie joue un rôle important dans le diagnostic. Le traitement est une urgence relative qui est essentiellement chirurgical. Le but de notre travail est de rappeler cette pathologie rare, qui passe souvent inaperçu et source d'handicap fonctionnel.

## Patient et observation

### Observation N^°^ 1

**Figure 1:**
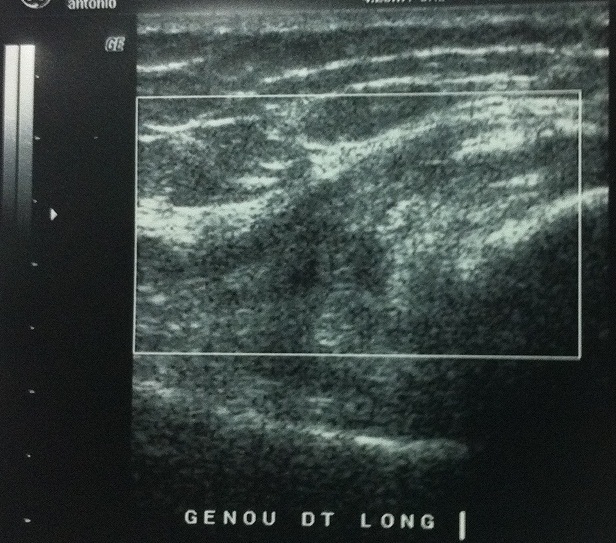
Echographie montrant rupture de la portion distale du tendon du muscle droit antérieur.

**Figure 2:**
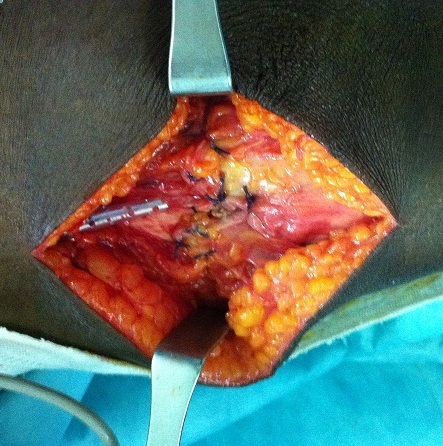
Suture termino-terminale par des points en U de sens opposes.

Il s'agit d'un homme de 50 ans, sportif occasionnel, sans antécédents pathologiques notables. Victime d'un accident de sport par chute avec genou en flexion contrarié ce qui a occasionné une douleur vive et une sensation de déchirure, suivi d'une impotence fonctionnelle partielle du membre inferieur droit. Le patient a consulté une semaine après le traumatisme. L'examen clinique a objectivé un vide suprapatellaire avec perte de l'extension active de la jambe sur la cuisse. Le bilan radiologique du genou droit a montré une rotule basse en faveur d'une rupture du tendon quadricipital. L’échographie et l'IRM du genou ont montré une rupture au niveau de la portion distale du tendon quadricipital (Figure 1). Le patient a été opéré après un mois du traumatisme, sous rachianesthésie avec un garrot pneumatique à la racine du membre inferieur droit. Une voie d'abord antérieure et médiane a été réalisée. Le geste chirurgical a consisté en la réalisation d'une suture termino-terminale avec des points en “U” de sens opposés par du fil Vicryl n^°^ 2, renforcé par un surjet avec le même type de fil (Figure 2). En postopératoire, les suites ont été simples sans accident thrombo-embolique ou sepsis. Le membre opéré a été immobilisé par une attelle maintenant le genou en extension pendant 6 semaines. La rééducation passive a été débutée précocement. La rééducation active n'a été autorisée qu’à la 6éme semaine. Après un recul de 6 mois, l'examen clinique a objectivé une flexion de 120^°^ avec une complète extension active et une force du quadriceps cotée à 4.

### Observation N^°^ 2

**Figure 3:**
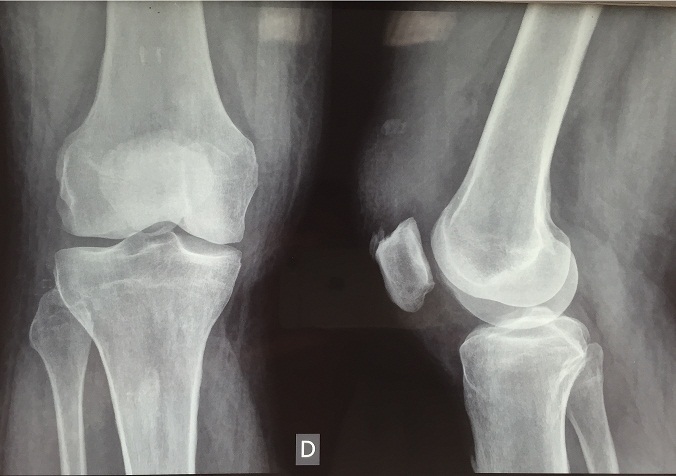
Radiographies standards du genou droit (face+profil) montre une rotule basse.

**Figure 4:**
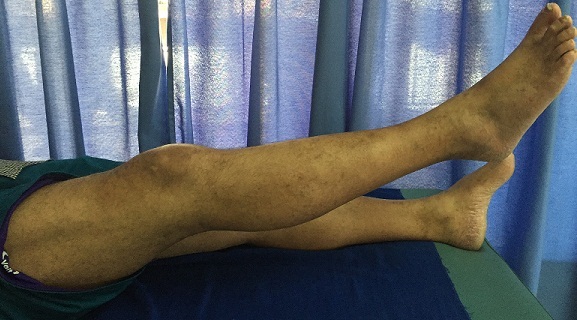
Contrôle clinique à 6 mois: extension complète après rééducation.

**Figure 5:**
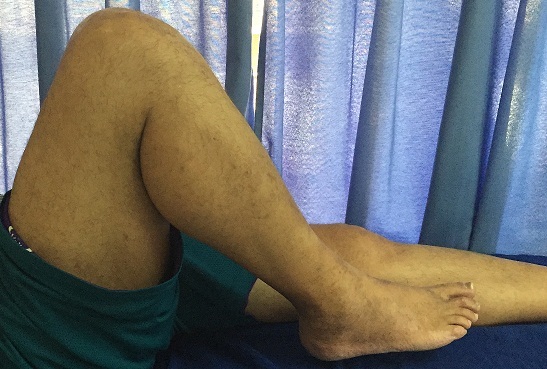
Contrôle clinique à 6 mois: flexion non limitée après rééducation.

**Figure 6:**
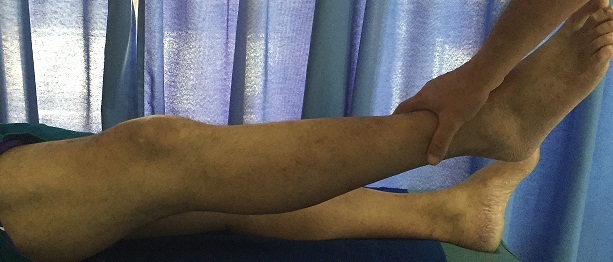
Contrôle clinique à 6 mois: force du quadriceps cotée à 4 après reeducation.

Un homme de 54 ans, sportif occasionnel, sans antécédents pathologiques notables. Victime lors d'un exercice de sport par des mouvements d'accroupissements et surélévations, d'une douleur vive et une sensation de déchirure, suivie d'une impotence fonctionnelle totale du membre inferieur droit. Le même jour du traumatisme, Le patient a consulté dans notre formation. L'examen clinique a objectivé un gonflement de la cuisse droite au niveau du ¼ inferieur. A la palpation, on a trouvé une fluctuation suprapatellaire et un choc patellaire, avec perte de l'extension active de la jambe sur la cuisse. Les radiographies standards du genou droit ont montré une rotule basse (Figure 3). L'IRM a confirmé le diagnostic de rupture du tendon quadricipital. Le geste chirurgical a été réalisé 2 semaines après le traumatisme, sous rachianesthésie avec un garrot pneumatique à la racine du membre. L'exploration opératoire a permis de confirmer la rupture en plein corps tendineux. La réparation chirurgicale a été faite, par la suture des berges tendineuses avec des points en U de sens opposés grâce au fil de type Vicryl n^°^ 2 et renforcée par du surjet. Les suites opératoires ont été sans événement particulier, le genou droit a été mis dans une attelle en légère flexion pendant 6 semaines. La rééducation en isométrique a été démarrée le deuxième jour, puis active après 6 semaines du geste chirurgical. Après 6 mois, le patient a été revu avec une extension totale du genou, une flexion dépassant 110° et une force du quadriceps cotée à 4 (Figure 4, Figure 5, Figure 6).

### Observation N^°^ 3

**Figure 7:**
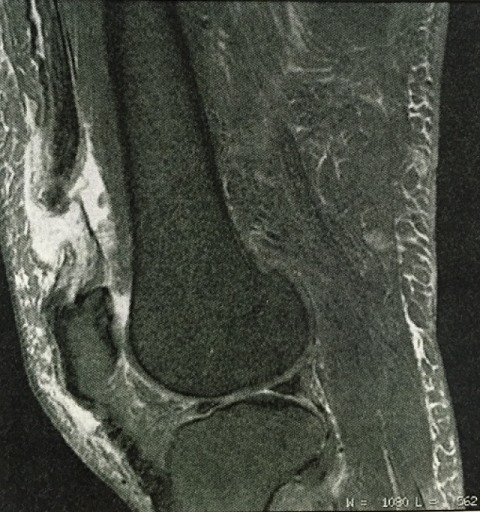
IRM montre une rupture quasi-totale du tendon quadricipital.

Il s'agit d'un homme de 48 ans, sans antécédents pathologiques notables. Victime d'une chute dans les escaliers avec un membre inférieur droit en extension et le pied droit bloqué au sol, ce qui a entraîné une douleur aiguë et une sensation de déchirure, suivie d'une impotence fonctionnelle partielle. Le patient a consulté le même jour aux urgences d'une autre structure. Il a été traité comme une entorse du genou droit. 3 semaines après, le patient a consulté dans notre formation. Il était obèse (110 kg). L'examen physique a objectivé un gonflement de la cuisse droite au niveau du ¼ inférieur avec boiterie à la marche. Une douleur et un vide sus rotulien sont révélés à la palpation. L'IRM a été réalisée et a objectivé une interruption de la continuité du tendon quadricipital droit avec une plage en hypersignal, de tonalité presque liquidienne, s'interposant entre les deux fragments tendineux qui sont eux en hyposignal, plus ou moins remaniés et effilochés (Figure 7). Le patient a bénéficié de la même technique chirurgicale sous rachianesthésie avec garrot pneumatique à la racine du membre inférieur droit. Une voie d'abord antérieure et médiane a permis de réaliser une suture termino-terminale grâce à des points en U de sens opposés par du Vicryl n^°^ 2 et renforcés par du surjet. Une immobilisation postopératoire par plâtre cruropédieux en légère flexion a été faite pendant 45 jours. La rééducation isométrique a débuté précocement et l'active après 6 semaines. Le patient a été revu au 6^éme^ mois. Il a présenté une plainte fonctionnelle qui a été modérée et compatible avec son activité habituelle (douleur du genou avec une gêne dans les escaliers et à la marche). A l'examen clinique, la flexion du genou a été chiffrée à 110^°^ avec un déficit d'extension active de 7^°^ et la force du quadriceps a été cotée 3. Au recul de 16 mois, les résultats ont été très bons dans les deux premiers cas et bon dans le troisième cas. Ils ont repris leur activité sportive.

## Discussion

Le tendon quadricipital se constitue de quelques centimètres seulement au dessus de la rotule par les terminaisons des tendons du quadriceps. Sa rupture intéresse les 4 composants et se situe à environ 1 à 2 cm au dessus du bord supérieur de la rotule. La vascularisation des fibres tendineuses antérieures s’étend de la jonction musculo-tendineuse jusqu’à l'insertion rotulienne. Au niveau de la couche profonde, il existe une plage avasculaire. Cette zone pourrait donc expliquer la survenue de lésions dégénératives, plus fréquentes sur les faisceaux moyens et postérieur hypo- vascularisés, d'autant plus que les contraintes sur la face postérieure du tendon sont majorées lors de l'hyperflexion du genou qui plaque ces éléments postérieurs contre la trochlée [3].

La rupture du tendon quadricipital est une affection rare parmi les lésions traumatiques du système extenseur du genou. Elle vient après les fractures de la patella et la rupture du ligament patellaire. Elle Touche le plus souvent le sujet sportif plus âgé après 40 ans [4]. Plusieurs facteurs prédisposent à cette lésion tendineuse en particulier: l’état de fatigue générale avec absence de récupération musculaire et après des efforts soutenus; le froid ou la déshydratation chez des patients corpulents sinon obèses [5]; les pathologies préexistantes telle les maladies de système (la polyarthrite rhumatoïde, le diabète, le lupus érythémateux, l'hyperparathryroïdisme); l'affaiblissement iatrogène du tendon suite à un prélèvement chirurgical pour plastie (Mac Intoch) [4]; la rupture tendineuse après prothèse du genou avec médaillon rotulien est également fréquente, à cause de l'amincissement de la zone d'insertion ou la fragilisation de cette dernière du fait de la réduction de l’épaisseur de la rotule et de la dévascularisation de la zone d'attache [6].

Le mécanisme est le plus souvent indirect par contraction brutale de l'appareil extenseur afin d’éviter la chute [7]: soit au cours d'une réception brutale après un saut, à cause des contraintes de freinage brusque imposées au quadriceps à cette occasion; soit lors d'une hyperflexion brutale du genou lors d'une chute. Le traumatisme direct est reconnu mais il est rare. Cliniquement, l'interrogatoire permet d'orienter vers le diagnostic. Il se manifeste par une douleur vive et intense. L'impotence fonctionnelle peut être complète ou remplacée par une simple gêne à la marche. La palpation retrouve une dépression sus-rotulienne et perceptible à la palpation, alors que l'hémarthrose peut être absente. Il y a de toute façon un empâtement volumineux sus-rotulien, qui doit faire évoquer le diagnostic et rechercher le déficit d'extension. Devant ce tableau clinique, il faut penser à ce type de lésion. 40% des cas de rupture du tendon quadricipital sont initialement non diagnostiquées selon Bianchi [8]. Il faut demander une imagerie dès qu'on y pense afin d'affirmer ou d’éliminer cette pathologie au moindre doute.

Le bilan radiographique conventionnel est réalisé systématiquement en cas de doute et pour rechercher une rotule basse, basculée dans le plan sagittal, et aussi permet d’écarter le diagnostic de fracture de la patella. L’échographie suprapatellaire est un examen rapide et fiable qu'il faut penser à demander. Il peut objectiver une zone hypoéchogène traversant toute l’épaisseur du tendon, et un épaississement intra-tendineux. Elle se fait en légère flexion ce qui permet habituellement de faciliter le diagnostic en favorisant un discret élargissement de l'espace inter-fragmentaire, elle est plus performante que l'IRM au stade aigu et subaigu. L'IRM est un examen déterminant surtout dans les formes anciennes [9]. Il est le plus performant, et montre une disparition des fibres tendineuses et la présence de remaniements oedémato-hémorragiques [9].

L’évolution en l'absence de traitement se fait vers la cicatrisation en rétraction, laissant persister une dépression et une tuméfaction contractile à la partie inférieure de la cuisse. Le traitement est essentiellement chirurgical. Sa précocité donne des meilleurs résultats dans les ruptures totales aiguës et doit être la règle. Les lésions négligées ou découvertes secondairement doivent aussi bénéficier d'une réparation chirurgicale. On procède par une voie d'abord antérieure médiane avec prolongement de 8 cm environ au dessus du bord supérieur de la patella vers le haut, et jusqu'au bord inférieur de la patella vers le bas. Ensuite, une incision du fascia permet l’évacuation de l’épanchement. Il est recommandé de faire un prélèvement pour l’étude histologique à la recherche d'une pathologie favorisante [5]. La réparation est obtenue par la remise en contact des bouts tendineux par une rangée de point en « U ». Cette suture peut être renforcée par une plastie soit de type passage du semi tendineux pour assurer la protection biologique de la suture [10], soit aussi par l'utilisation d'une bandelette de tendon rotulien et surtout fibreux retournée vers le haut selon Ait Si Selmi et al [11].

Une immobilisation en légère flexion est indispensable pendant 45 jours pour éviter au patient comme au kinésithérapeute de mettre en danger la suture. La reprise de l'appui est immédiate, sous couvert de béquilles. La rééducation est ainsi commencée dès les premiers jours [9]. Une mobilisation passive précoce est préconisée dans un secteur de 0 à 60^°^ de flexion, puis rééducation active à partir de la 5^ème^ ou 6ème semaine, comprenant la récupération de la mobilité articulaire en flexion, le travail de l'extension active et la récupération de la trophicité et la force du quadriceps. Les séries de la littérature concluent à la fréquence des bons et très bons résultats fonctionnels après traitement chirurgical d'une rupture du tendon quadricipital associé à une immobilisation et rééducation fonctionnelle précoce [2, 12].

## Conclusion

Les ruptures du tendon quadricipital se rencontrent essentiellement chez le sportif au-delà de 40 ans. Le diagnostic doit être précoce pour optimiser la prise en charge en milieu orthopédique. L’échographie reste l'examen de base si le diagnostic est évoqué précocement. Un complément par l'IRM est indispensable si l’échographie est insuffisante surtout dans les formes anciennes. La prise en charge chirurgicale doit être précoce, seul garant d'une récupération fonctionnelle complète. En cas de retard diagnostique, même si les patients sont peu symptomatiques, le résultat à long terme est moins bon avec un déficit d'extension active plus important et une force du quadriceps moindre.
